# Extracellular Matrices to Modulate the Innate Immune Response and Enhance Bone Healing

**DOI:** 10.3389/fimmu.2019.02256

**Published:** 2019-09-20

**Authors:** Andrés García-García, Ivan Martin

**Affiliations:** Tissue Engineering, Department of Biomedicine, University Hospital Basel, University of Basel, Basel, Switzerland

**Keywords:** tissue engineering, extracelullar matrix, immunomodulation, bone repair, innate immune system, mesenchymal stromal cell, regenerative medicine

## Abstract

Extracellular matrices (ECMs) have emerged as promising off-the-shelf products to induce bone regeneration, with the capacity not only to activate osteoprogenitors, but also to influence the immune response. ECMs generated starting from living cells such as mesenchymal stromal cells (MSCs) have the potential to combine advantages of native tissue-derived ECMs (e.g., physiological presentation of multiple regulatory factors) with those of synthetic ECMs (e.g., customization and reproducibility of composition). MSC-derived ECMs could be tailored by enrichment not only in osteogenic cytokines, but also in immunomodulatory factors, to skew the innate immune response toward regenerative processes. After reviewing the different immunoregulatory properties of ECM components, here we propose different approaches to engineer ECMs enriched in factors capable to regulate macrophage polarization, recruit host immune and mesenchymal cells, and stimulate the synthesis of other immunoinstructive cytokines. Finally, we offer a perspective on the possible evolution of the paradigm based on biological and chemico-physical design considerations, and the use of gene editing approaches.

## Introduction

Bone disorders have a worldwide prevalence since they can be derived from multiple causes, including orthopedic trauma, cancer or congenital diseases. Since it emerged in the early 90s, bone tissue engineering has aimed to develop innovative biological materials to improve bone repair and regeneration (1, 2). Among different biomaterials, extracellular matrices (ECMs) have been proposed as one of the best candidates to fabricate grafts for bone regeneration (3). Native tissue-derived ECMs represent a physiological solution providing not only structural support, but also multiple biomolecules capable to modulate the behavior of both resident and recruited cells in the context of bone healing (4–6). However, they exhibit limited reproducibility in their composition, can lead to pathogen transmission and lack the possibility of customization. Furthermore, native ECMs are rich in immunogenic molecules that can trigger an uncontrolled response and affect graft integration (7). Synthetic ECMs, typically in the form of hydrogels, have been developed as tunable alternatives, with promising results also in the context of bone repair (8). However, they still rely on the presentation of a limited set of signals, in ways which do not entirely recapitulate physiological processes. ECMs could be also generated from living cells, e.g., mesenchymal stromal cells (MSCs), using typical tissue engineering paradigms, and afterwards decellularized (9, 10). The resulting ECMs would in principle combine the advantages of a physiological system with the possibility of standardization (e.g., through the use of immortalized cell lines) and tunability (e.g., by genetic modification of the cells used) (11). Decellularization of the MSC-generated ECMs can also be designed to improve the immunogenic properties of the resulting material (12, 13).

Despite many advances, the need for quality improvement of engineered ECM (either synthetic or MSC-generated) for bone healing is still quite large (3). Along this line, ECMs might be enriched in morphogens or angiogenic factors to enhance bone regeneration. Importantly, multiple evidences have revealed that a proportionated and coordinated immune system response is essential to critically promote bone healing. Indeed, many studies in the past years have revealed a broad crosstalk between the skeletal and immune systems through many shared cytokines, molecular pathways and transcription factors. All these findings have contributed to define the so-called osteoimmunology field, in which engineering ECMs to modulate immune signals has become one of the spearhead (14). In this context, current strategies do not aim to suppress the immune response, but rather engineer ECM-derived materials to present osteoimmunomodulatory factors and instruct the inescapable immune response in favor of bone regeneration (15).

In this review, we describe firstly key aspects of the interplay between innate immunity and bone healing. Then, we highlight how some ECM components are able to modulate the innate immune response. Finally, we summarize different strategies proposed for ECMs enriched with innate immunoinstructive factors to improve bone regeneration.

## Innate Immune System in Bone Repair

All bone substitute materials, as any other foreign structure, trigger a host immune reaction after implantation, which recapitulates the first steps of the classical immune response after bone injury (16, 17). In addition, implantation surgery is not more than a controlled injury. Therefore, understanding the immune cascade following bone injury is key to generate immunoinstructive scaffolds capable to enhance bone regeneration.

Immediately after any bone injury, vascular disruption generates a hematoma and triggers a quick and potent inflammatory reaction. Multiple blood and interstitial fluid proteins [e.g., Factor XII and tissue factor (TF)] adsorb the injury site and activate the blood coagulation cascade as well as the complement system (18). In this context, activated platelets play a critical role producing prothrombinases, which activate thrombin serin protease and allow the amplification of the coagulation process (19). All these proteins lead to a transient fibrin clot formation that constitutes the matrix for the recruitment of the first immune cells. In contrast with later stages, the onset of the acute inflammatory response is mostly governed by the innate immune system, whose main players are polymorphonuclear leukocytes (PMNs, neutrophils), monocytes and tissue-resident macrophages (20).

Circulating PMNs are quickly recruited by this chemoattractant protein matrix to the injury site. While they might contribute to fibrin clot formation (21), their main roles involve the release of proteolytic enzymes to promote tissue remodeling, and inflammatory cytokines (such as IL1β, TNFα, IL8, MCP1, or MIP1β) to recruit other myeloid cells and MSCs (15). Recruited monocytes release more cytokines and differentiate into macrophages. Both monocytes derived- and tissue resident macrophages have been revealed essential for successful bone formation (22). The relevance of this cell type resides in its capacity to exhibit different functional phenotypes in response to environmental cues (23). Initially, the inflammatory storm upon bone injury polarizes macrophages toward an activated M1 phenotype. M1 macrophages release more inflammatory cytokines to contribute to cell recruitment and dead cell clearing. At later stages, macrophages are alternatively polarized toward an anti-inflammatory M2 phenotype. These cells secrete tissue repair factors (IL10, IL1ra, TGFβ1, or VEGFα) to resolve the inflammation, recruit MSCs, promote angiogenesis and induce endochondral bone formation (24). Recruited MSCs undergo chondrogenic differentiation adjacent to the fracture site to form bone by endochondral ossification, while direct intramembranous ossification takes place under the periosteum (25). Interestingly, they also play a crucial paracrine role releasing immunosuppressive cytokines to resolve site inflammation. Human MSCs suppress innate immune cells migration, proliferation and differentiation through multiple pathways including Notch and PGE-2 signaling (26). Therefore, the coordinated crosstalk between MSCs/osteoprogenitor cells and macrophages is critically required for successful bone healing.

Following these principles, several studies have attempted to improve bone regeneration modulating either macrophage number or their polarization toward M1 or M2 phenotypes (27). On the one hand, it has been reported that the expression of some pro-inflammatory signals right after injury significantly improves bone healing. As examples, TNFα promotes postnatal intramembranous bone repair through the induction of osteoprogenitor cell recruitment or osteogenic cell activation (28), while IL1β administration could favor endochondral bone formation after injury (29, 30). Similarly, IL-6 family signaling was shown to stimulate bone formation during the inflammatory process (31). On the other hand, different studies have proposed that an anti-inflammatory M2 environment is more suitable for human MSC activity (32) and delivers osteoinductive signals (33). In this regard, IL4 administration could decrease bone degradation after joint replacement (34).

Accumulating evidences suggest that an appropriate transition from the inflammatory M1 to the anti-inflammatory M2 phenotype favors bone regeneration by endochondral ossification (24, 35). However, macrophage activation and polarization are very complex *in vivo*, since the exposition to multiple signaling leads to activation of macrophages with mixed functions. This is especially prominent in pathological conditions, where abnormal signaling might prime macrophages toward a profibrotic phenotype (36). Indeed, macrophage activation nomenclature has been recently revised to unify criteria for the diverse experimental scenarios (37).

In the context of ECM engineering, some researchers have used myeloid cells to improve ECM-derived grafts integration after implantation and/or promote bone healing after trauma or bone degeneration. Although the supplementation of ECM-derived grafts with peripheral blood monocytes did not seem to increase bone regeneration by itself (38), peripheral blood-derived macrophages were reported to be essential in the degradation and remodeling of ECM-based materials (39). Other studies have developed strategies to generate immunoinstructive ECMs by modulating macrophage polarization during bone healing and promote bone formation (40). However, the success of these approaches is often subjected to several variables like patient health, trauma size or ECM composition.

## ECM Composition and Innate Immunity

Many endogenous ECM components exhibit important immunomodulatory features that can decisively influence the innate immune response *in vivo* (41, 42). For example, the collagenous network is, together with the proteoglycans, the main component of bone tissue ECM that defines its mechano-physical features. However, collagen fibers exhibit motifs that can interact with some immune cell receptors. In particular, macrophages can specifically adhere to denatured forms of collagen type I fibers through their scavenger receptors (43). Furthermore, collagen fibers have been reported to affect metalloproteinase 9 (MMP9) secretion on the macrophage-like U937 cell line (44).

Hyaluronic acid is one of the most important glycosaminoglycan of native ECMs and it has been proposed to play a dual immunomodulatory role based on its molecular weight. Whereas, intact high molecular weight hyaluronic acid has a prominent anti-inflammatory effect inducing IL10 production by macrophages, damaged low molecular weight hyaluronic acid promotes a pro-inflammatory phenotype stimulating TNFα expression (45). Interestingly, this immune cells-hyaluronic acid crosstalk seems to be bidirectional, since monocyte activation can modulate its binding to hyaluronic acid too. More specifically, TNFα promotes monocytes-hyaluronic acid interactions through CD44 receptor, while IL4 administration is sufficient to abrogate this effect (46). Heparan sulfate, another important glycosaminoglycan that binds to ECM proteins to form proteoglycans, can also interact with the immune system to regulate cell adhesion, the availability of immune cytokines and leukocyte migration (47).

Importantly, not only components of native ECM have been reported to modulate the innate immunity. Fibrin is a molecule often used to build synthetic ECMs, which has been also shown to modulate macrophages behavior. This protein derives from fibrinogen after thrombin proteolytic activity and it is involved in the hemostatic clot formation after injury (48). Several studies have reported that fibrin could facilitate or block macrophages migration depending on its abundance in the matrix (49), and inhibit their pro-inflammatory properties (50). In contrast, fibrin degradation products induce leukocyte recruitment (51) and promote pro-inflammatory (IL1β, IL6) cytokines secretion by monocytes *in vitro* (52).

ECMs can also contain cryptic domains very similar to immune cytokines that are only exposed after proteolytic activity by metalloproteinases. In non-physiological conditions, the aberrant expression of these domains by exacerbated tissue remodeling can influence immune cell activation and survival (53, 54). Moreover, the decellularization step followed to generate non-immunogenic off-the-shelf grafts could also condition the immunomodulatory properties of ECM components. Pioneering work from Badylak using the bladder system showed that decellularized grafts preferentially induce an anti-inflammatory macrophage polarization, while cellular components trigger a pro-inflammatory polarization (55, 56).

Furthermore, different types of ECMs seem to induce a different innate immune response *in vivo*. For example, decellularized bone-derived ECM has a higher capacity to induce monocytes recruitment than cardiac ECMs, which might reflect the differential molecular composition of these matrices (57).

In summary, ECMs exhibit intrinsic immunomodulatory features which are mostly determined by their molecular composition. Therefore, a precise knowledge of the components of ECMs is essential to further develop their immunomodulatory properties with extrinsic factors.

## Exogenous Delivery of Specific Immunoregulators in Engineered ECMs to Modulate the Innate Immune Response

In order to modulate the innate immune response upon implantation, pro-inflammatory or anti-inflammatory cytokines can be directly delivered into the grafts. To antagonize the pro-inflammatory effect of IL1β, inhibitors of IL1R1/MyD88 signaling were covalently cross-linked into fibrin matrix to improve MSC-based bone regeneration in mice (58).

Immune cytokines could be also delivered sequentially in order to facilitate the transition between the inflammatory and anti-inflammatory phases during bone healing. For instance, Spiller et al. physically adsorbed IFNγ onto the scaffolds and attached IL4 using biotin-streptavidin binding to drive the sequential polarization of macrophages from M1 to M2 phenotype. These scaffolds also exhibited increased vascularization upon *in vivo* implantation, which proved their functionality (59). Along the same line, another study confirmed that IL4 released from a nanometer-thickness coating is critical promoting the M1-to-M2 transition during bone tissue repair and improving implant integration (60). Recently, Schlundt et al. further demonstrated the importance of M2 macrophages to induce endochondral ossification in the context of bone healing. Indeed, they added IL4 and IL13 to the collagen scaffolds prior to insertion in an osteotomy model. In this way, they stimulated M2 macrophage polarization and improved bone regeneration (24).

In addition to interleukins, synthetic peptides represent an alternative way to modulate the immunomodulatory features of ECMs. The peptide Arg-Gly-Asp (RGD), contained in basement membranes components such as entactin or presented in photopolymerizable poly(ethylene glycol) (PEG)- based hydrogels, has been shown to enhance myeloid cells adhesion to the ECM (61, 62), while it induces macrophage polarization toward an anti-inflammatory profile via integrins interactions (63). As another example, a synthetic peptide binding to LAIR1, a receptor expressed in multiple immune cells, has been reported to reduce pro-inflammatory cytokines release by BM-derived macrophages. Interestingly, this effect was only observed when the peptide was linked to the scaffold surface (64). On the other side, TP508, a synthetic 23-aminoacid peptide representing a receptor-binding domain of human thrombin, promotes bone healing in a rat femoral fracture model by inducing inflammatory mediators release and angiogenesis (65). Adsorbed fibrinogen or scaffolds made of this material could also elicit a favorable immune response and improve the osteogenic capacity in a critical size bone defect in rats (66, 67). Among lipid compounds, specific prostaglandin agonists administration could enhance bone formation after injury avoiding systemic inflammation induction (68, 69). For example, prostaglandin E EP4 receptor agonist was shown to synergize with BMP2 and activate osteoprogenitor cells when delivered in a biodegradable copolymer composed by poly-D,L-lactic acid with random insertion of p-dioxanone and polyethylene glycol (70).

The anti-inflammatory properties of glucocorticoids are well-known. In particular, dexamethasone delivery in polydimethylsiloxane-based 3D scaffolds has been used to promote macrophage polarization toward an anti-inflammatory (M2) phenotype and suppress inflammatory pathways during the first week post-implantation (71). Dexamethasone delivery using poly (lactic-co-glycolic acid) microsphere/polyvinyl alcohol hydrogel composites has been shown to elicit an anti-angiogenic effect which could be overcame by co-administering VEGF (72).

Different approaches have been here discussed to deliver immunoregulatory factors into ECM in order to instruct the innate immune response *in vivo*. Nevertheless, the delivery of exogenous factors is subjected to several drawbacks including poor matrix penetration, diffusion, enzymatic degradation and thus uncontrolled doses. In addition, the delivery of few specific agents has been revealed inefficient in triggering a complete immune response *in vivo*. For this reason, different strategies have been developed to control the spatial and temporal delivery (73–75). Among them, 3D multilayer systems and intelligent hydrogels have been tested for the sequential release of several factors to ECM-based scaffolds (76, 77). Biomimetic biomaterials, like hydrogels, have been developed to achieve a molecular-level modulation. This includes strategies to immobilize incorporated factors by cross-linking and approaches based on protease-dependent degradation to release them (78). Other options to engineer immunoinstructive ECMs directly target MSCs or immune cells to modulate the natural production and release of immune factors by these cells.

## ECM-Driven Endogenous Synthesis of Immunoregulators by Host Cells to Modulate the Innate Immune Response

Aiming to generate ECM grafts instructed to trigger a more physiological immune response, many researchers have tried to use several biological agents to stimulate host MSCs and/or immune cells to deliver key immune cytokines and enhance bone formation. Macrophage recruitment is critical for dead tissue clearance and modulate the inflammatory cascade in bone healing. Kim et al. used a sphingosine-1 phosphate agonist in combination with platelet-rich plasma to sequentially induce pro-inflammatory (TNFα) and anti-inflammatory (OPG, IL10, and TGFβ1) signals in order to promote macrophages recruitment and enhance bone healing (79). In contrast, adding high sulfated hyaluronan to collagen I-enriched ECMs impairs the secretion of IL1β, IL8, IL12, and TNFα, while it enhances the production of IL10 and CD163 expression in macrophages (80).

Interestingly, inorganic compounds like magnesium-doped calcium phosphate cement are also able to elicit a favorable innate immune reaction modulating macrophage activity to improve osteogenesis and angiogenesis. This compound represses TNFα and IL6 expression while it upregulates TGFβ1 in macrophages (81). Beyond macrophage activation, immunoregulators have been also used to modulate MSC behavior. For example, the combination of RGD peptide and 3D hyaluronic acid hydrogels can influence MSC integrin expression (82).

To sum up, these studies attempt to improve bone regeneration by targeting endogenous MSC/immune cells to produce themselves the cues critical for an orchestrated repair upon bone injury (83).

## Conclusions and Perspectives

In this work, we have reviewed some relevant aspects of the interplay between the innate immune system and osteogenesis in the context of bone healing. Then we have focused on the interactions between ECM components and innate immune cells to finally discuss some strategies followed to immune-instruct ECMs. However, many other critical aspects have not been discussed here.

As previously mentioned, the innate immunity plays an essential role during the initial phases after bone injury, promoting cell immunorecruitment and modulating the inflammatory environment (M1-to-M2 paradigm). Importantly, the adaptive immune response takes slowly part in this regulation to instruct the bone formation phase. Multiples studies have attempted to engineer ECM-based materials to modulate the adaptive immune response, specially targeting T cells (84). Indeed, many efforts are currently conducted to better coordinate the activity of both branches of the immune response after engineered graft implantation.

We have discussed how different ECM components, even in the absence of immunomodulatory factors, could modulate the innate immune response. The works studying these interactions reveal that ECM composition is an important factor to consider prior to any further immunoregulatory engineering. However, beyond its chemical properties, ECM physical features can also decisively modulate the immune response *in vivo* (85). Therefore, strategies to enrich ECMs in immunoinstructive factors should be coupled with the engineering of endogenous physical and chemical properties of the ECM used (86).

Different approaches have been proposed to improve the spatiotemporal delivery of growth factors to engineer “smart” ECMs. However, in most cases they only focus on osteogenic and angiogenic factors. Immunomodulatory ECM-like microspheres have been recently used to improve IL4 delivery and accelerate bone regeneration modulating macrophage polarization (87). Future studies should aim to a coordinated delivery of osteogenic, angiogenic and immune factors according to the natural stages of bone healing.

**Figure 1:**
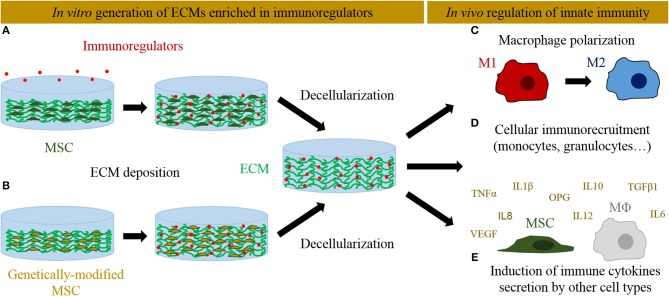
Different *in vitro* approaches followed to deliver immunoregulators into ECM-derived scaffolds and their interactions with the innate immune system *in vivo*. **(A)** Immunoregulators can be directly supplemented and anchored into MSC-derived ECMs. **(B)** Alternatively, MSCs can be genetically edited to overexpress immunoregulators and seeded on scaffolds, where they will produce an ECM enriched in those factors. The tissues are later decellularized to generate cell-free ECMs. *In vivo*, these immunoinstructive ECMs can activate innate immunity at different levels: **(C)** induce macrophage polarization toward an anti-inflammatory M2 phenotype, **(D)** recruit immune cells, and **(E)** induce the secretion of immune cytokines by recruited mesenchymal stromal cells and macrophages (MΦ).

Genetic manipulation of MSCs has also emerged as an alternative to better control the dose and temporal delivery of osteogenic and angiogenic factors into engineered ECMs to improve bone regeneration process (88). Genetically modified MSCs could contribute directly to bone formation promoting osteoprogenitor cells differentiation, but also indirectly enhancing host cells recruitment. The most followed approaches involve the expression of the osteoinductive bone morphogenetic protein (BMP) family factors to stimulate bone repair. In particular, BMP2-overexpressing cells have been successfully used to speed up the repair of critical-size bone defects in rodent models (89, 90). Other overexpressed factors like Osterix aimed to induce osteogenic differentiation (91, 92). As a master regulator of angiogenesis, VEGF has been overexpressed in different cell types to favor tissue vascularization (93). A VEGF-overexpressing MSC line gives rise to ECMs with high VEGF content and superior vasculature in an ectopic implantation model (11). In addition to its angiogenic properties, VEGF could also modulate the immune response (94). Similarly, sphingosine 1-phosphate has been reported to enhance vascularization and bone formation (95), but at the same time it also plays multiple roles in the innate immunity (96). These works represent examples of how MSC can be genetically engineered to generate ECMs enhancing osteogenesis and vasculogenesis. An analogous approach could be pursued to overexpress specific osteoimmunomodulatory factors and thus generate immunoinstructive ECMs (Figure 1). In this context, MSCs overexpressing IL4 and IL10 have been proposed as promising tools to mitigate chronic inflammation diseases (such as arthritis) and promote tissue regeneration (97, 98). However, their capacity to generate immunoinstructive ECMs have not been yet explored. Moreover, the development of inducible cell lines might represent an interesting refinement to control the temporal expression of these key genes (98). Delivering candidate genes efficiently into the cells without viral vectors (which may carry safety concerns) remains an open challenge (99).

In summary, important advances have been achieved in the last years to improve the quality of immunoinstructive ECM-derived grafts and their immunogenicity after implantation. In the context of ECM engineering, immunoregulators can be exogenously delivered to enrich the biomaterial in specific cytokines and/or stimulate the endogenous synthesis of other factors by host cells. In this perspective, genetically modified MSCs represent a relevant alternative to control the spatiotemporal delivery of immunoregulators in order to engineer immunoinstructive ECMs promoting efficient bone repair.

## Author Contributions

AG-G and IM prepared the figures and wrote the manuscript.

### Conflict of Interest

The authors declare that the research was conducted in the absence of any commercial or financial relationships that could be construed as a potential conflict of interest.
